# Association between ERCC2 Lys751Gln polymorphism and the risk of pancreatic cancer, especially among Asians: evidence from a meta-analysis

**DOI:** 10.18632/oncotarget.15394

**Published:** 2017-02-16

**Authors:** Yang Wu, Zi-Peng Lu, Jing-Jing Zhang, Dong-Fang Liu, Guo-Dong Shi, Chun Zhang, Zhi-Qiang Qin, Jian-Zhong Zhang, Yuan He, Peng-Fei Wu, Yi Miao, Kui-Rong Jiang

**Affiliations:** ^1^ Pancreas Center, The First Affiliated Hospital of Nanjing Medical University, Nanjing, China; ^2^ Pancreas Institute, Nanjing Medical University, Nanjing, China; ^3^ Department of Digestive Diseases, Songjiang Branch Hospital of Shanghai First People's Hospital, Nanjing Medical University, Shanghai, China; ^4^ Department of Urology, The First Affiliated Hospital of Nanjing Medical University, Nanjing, China; ^5^ Department of Gastrointestinal Surgery, Huai'an Affiliated to Xuzhou Medical University and Huai'an Second People's Hospital, Huai'an, China

**Keywords:** pancreatic cancer, ERCC2, rs13181, polymorphism, meta-analysis

## Abstract

Single nucleotide polymorphisms (SNPs) of Excision repair cross-complementing group 2 (ERCC2) gene are suspected to affect the risk of pancreatic cancer. Many studies have reported the association between ERCC2 Lys751Gln polymorphism (rs13181) and the susceptibility to pancreatic cancer, but the outcomes remained controversial. To comprehensively determine this association, we conducted a meta-analysis based on a total of eight studies. Evidence for this association was obtained from the PubMed, EMBASE, Web of Science and Chinese National Knowledge Infrastructure (CNKI) databases. In general, a significant association was found between ERCC2 rs13181 polymorphism and the susceptibility to pancreatic cancer in four genetic models [CC vs. AA: OR = 1.56, (95% CI: 1.28-1.90), P = 0.470; AC/CC vs. AA: OR=1.20, (95% CI: 1.06-1.36), P = 0.396; CC vs. AC/CC: OR = 1.50; (95% CI: 1.24-1.81), P = 0.530; C vs. A: OR=1.22, (95%CI:1.11-1.34), P = 0.159]. Furthermore, stratified analyses by ethnicity indicated a significant association only in the Asian population. Our results indicate that the ERCC2 Lys751Gln polymorphism might be important in stimulating the development of pancreatic cancer, especially for Asians.

## INTRODUCTION

As a highly lethal disease, Pancreatic cancer is correlated with a very poor prognosis, characterized by the close parallel between incidence and mortality [1]. In the United States, five-year survival rate in pancreatic cancer patients remains as low as 6% [2]. The low survival rate is attributed to several factors, of which perhaps the most important is the late stage at which most patients are diagnosed. However, there is still no standard program for screening patients at high risk of pancreatic cancer and the accurate genetic epidemiology of this cancer remains unknown. As a multi-factorial disease, many factors are known to play a key role in pancreatic cancer development, such as smoking, obesity, drinking, diabetes as well as environmental chemicals [3–5]. Nevertheless, even when individuals are exposed to similar risk factors, not all of them develop into pancreatic cancer, which indicate that hereditary factors might play an essential role in pancreatic carcinogenesis.

According to the genetic profiles of pancreatic cancer, genomic instability mediated by DNA repair deficiency is a vital event in development of pancreatic carcinoma. DNA repair machinery plays a crucial role in defending cells against environmental hazards like ionizing radiation, ultraviolet (UV) rays, diet and smoking. As a key DNA repair mechanism, Nucleotide excision repair (NER) can influence gene-gene rearrangement, deletion, translocation and amplification [6, 7]. Excision repair cross-complementation rodent repair deficiency group 2 (ERCC2), which locates on chromosome 19q13.3, is an important genetic complementation group encoding for proteins involved in the NER pathway and could reverse ionizing radiation-induced damage and DNA damage by chemotherapy [8, 9]. Polymorphism rs13181, located at position 751 in exon 23, is the most common polymorphism in the coding region of ERCC2 and characterized by an A > C substitution leading to a lysine (Lys) to glutamine (Gln) amino acid exchange [10].

There are many studies which focus on the relationship between this SNP and pancreatic cancer [11–18]. Jiao et al. (2007), Hocevar et al. (2014), Ying et al. (2015) and He et al. (2016) did not observe any significant association between rs13181 and the risk of pancreatic cancer [11–14]. However, McWilliams et al. (2008), Zhao et al. (2015), Yan et al. (2016) and Sileng et al. (2016) suggested that this polymorphism was associated with an increased susceptibility to pancreatic cancer [15–18]. Although these researches are all based on experiment results, their results are always inconsistent and the roles rs13181 plays in pancreatic cancer are still unclear. Therefore, there is a need to make it clear whether this polymorphism is associated with pancreatic cancer. In order to assess the real association, the latest and most convincing evidence was utilized in this meta-analysis. This is, to our knowledge, the first comprehensive meta-analysis concerning the association between ERCC2 rs13181 and the risk of pancreatic cancer.

## RESULTS

### Literature search and study characteristics

**Figure 1:**
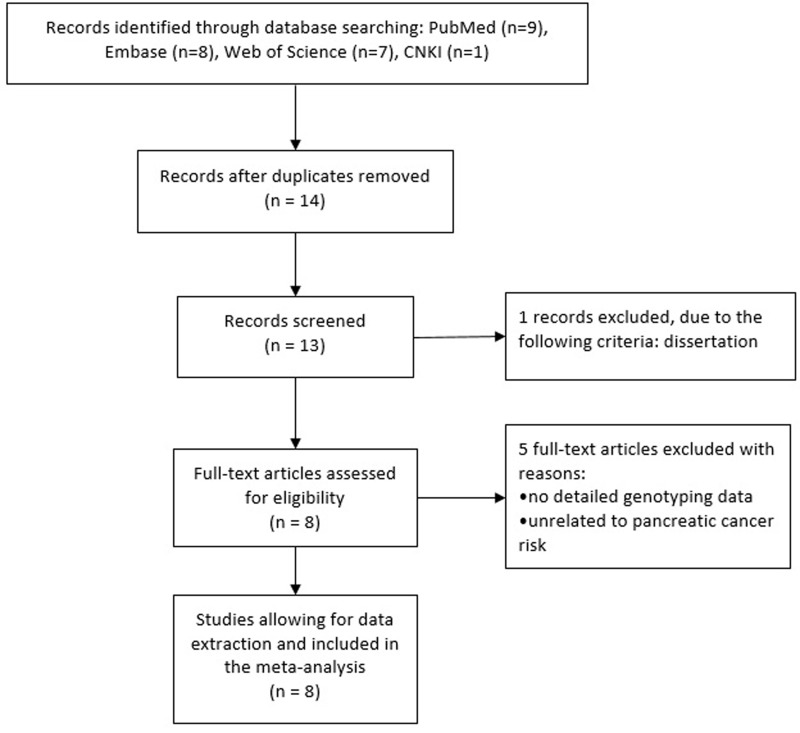
The flow diagram of retrieval for this study

The flow diagram of study exclusion and inclusion with specific reasons is shown in Figure 1. We identified 14 records, among which 8 papers appeared to be eligible for inclusion and were retrieved in full texts [11–18]. Among the six excluded articles, one was dissertation, four were not association studies on the risk of pancreatic cancer and one lacked sufficient data for estimating an odds ratio (OR) with 95% CI. Eventually, a total of eight case-control studies (1,980 cases and 2,317 controls) were ultimately included in the meta-analysis, and the details of each study were recorded in Table 1. As a result, each group of them was considered separately for pooling stratified analysis. These studies were divided into two groups based on the ethnicity of study participants, i.e., studies involving Caucasian population (3 studies) and those involving Asian population (5 studies).

**Table 1:** Characteristics of the 8 studies included in the meta-analysis

Author	Year	Ethnicity	Source of control^a^	Case			Control			Susceptibility^b^	*P* value of HWE^c^
				AA	AC	CC	AA	AC	CC		
He MG	2016	Asian	HB	119	78	20	143	86	15	N	0.668
Sileng A	2016	Asian	HB	116	103	35	138	121	18	Y	0.209
Yan D	2016	Asian	HB	118	70	38	167	65	31	Y	0.000
Ying MF	2015	Asian	PB	113	56	26	159	70	25	N	0.000
Zhao FL	2015	Asian	HB	131	72	43	159	64	23	Y	0.000
Hocevar AB	2014	Caucasian	HB	15	11	5	21	16	3	N	0.984
Mcwilliams RR	2008	Caucasian	PB	186	211	76	241	291	79	Y	0.544
Jiao L	2007	Caucasian	PB	124	184	30	147	203	32	N	0.001

^a^ HB, hospital-based studies; PB, population-based studies.

^b^ “Y” indicates an association between the rs13181 polymorphism and risk of pancreatic cancer; “N” means no association between rs13181 and the risk of pancreatic cancer.

^c^ HWE, Hardy–Weinberg equilibrium; P > 0.05 indicates that the participants in the control group met the HWE.

### Association between the rs13181 polymorphism and risk of pancreatic cancer

**Figure 2:**
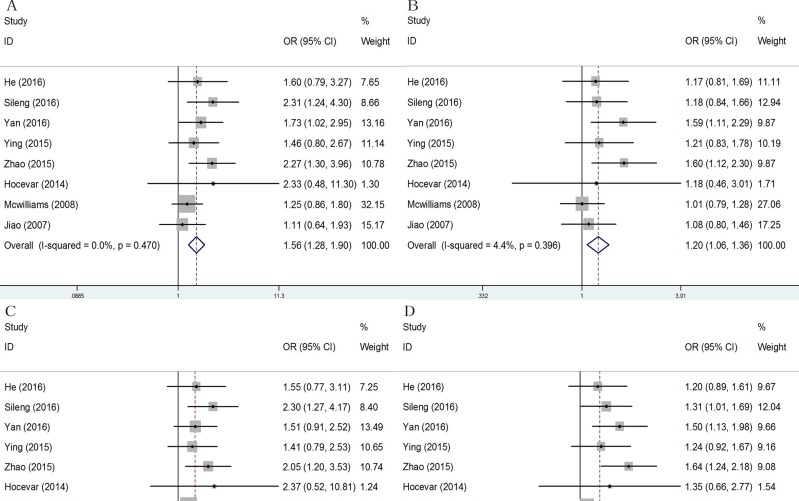
Forest plots of pancreatic cancer risk associated with ERCC2 rs13181 A > C polymorphism Four models showed statistical significance between ERCC2 rs13181 A > C and pancreatic cancer risk and the specific values were as follows. A. Homozygote model (CC vs. AA): OR = 1.56, 95% CI: 1.28-1.90; B. Dominant model (AC/CC vs. AA): OR = 1.20, 95% CI: 1.06-1.36; C. Recessive model (CC vs. AC/CC): OR = 1.50; 95% CI: 1.24-1.81; D. Allele model (C vs. A): OR = 1.22, 95% CI:1.11-1.34.

Forest plots of overall analyses with different models on the association between the rs13181 polymorphism and risk of pancreatic cancer are shown in Figure 2. All the results of overall and subgroup analyses are listed in Table 2.

**Table 2:** Meta-analysis results of association between rs13181 A > C polymorphism and pancreatic cancer risk

		AC *vs* AA		CC *vs* AA		AC/CC *vs* AA		CC *vs* AC/AA		C *vs* A	
Variables	*N*^a^	OR(95%CI)	*P*^b^/*I*^2^(%)	OR(95%CI)	*P*^b^/*I*^2^(%)	OR(95%CI)	*P*^b^/*I*^2^(%)	OR(95%CI)	*P*^b^/*I*^2^(%)	OR(95%CI)	*P*^b^/*I*^2^(%)
Total	8	1.10(0.96-1.25)	0.636/0.0	1.56(1.28-1.90)	0.470/0.0	1.20(1.06-1.36)	0.396/4.4	1.50(1.24-1.81)	0.530/0.0	1.22(1.11-1.34)	0.159/33.7
Ethnicity											
Asian	5	1.20(1.00-1.43)	0.584/0.0	1.87(1.43-2.43)	0.770/0.0	1.34(1.14-1.57)	0.534/0.0	1.74(1.35-2.25)	0.718/0.0	1.37(1.21-1.56)	0.507/0.0
Caucasian	3	0.99(0.82-1.21)	0.809/0.0	1.23(0.91-1.67)	0.681/0.0	1.04(0.86-1.25)	0.904/0.0	1.25(0.94-1.65)	0.583/0.0	1.07(0.94-1.23)	0.808/0.0
Source of controlc											
PB	3	1.02(0.85-1.22)	0.707/0.0	1.25(0.95-1.65)	0.803/0.0	1.07(0.90-1.27)	0.713/0.0	1.25(0.97-1.62)	0.758/0.0	1.09(0.96-1.24)	0.661/0.0
HB	5	1.20(0.99-1.46)	0.567/0.0	1.99(1.49-2.66)	0.895/0.0	1.36(1.14-1.62)	0.568/0.0	1.85(1.39-2.45)	0.809/0.0	1.40(1.22-1.61)	0.593/0.0
HWEd											
Yes	4	0.99(0.82-1.19)	0.939/0.0	1.52(1.14-2.01)	0.365/5.7	1.09(0.92-1.29)	0.853/0.0	1.53(1.17-1.99)	0.379/2.8	1.16(1.02-1.32)	0.618/0.0
No	4	1.23(1.02-1.49)	0.535/0.0	1.60(1.21-2.11)	0.339/10.7	1.33(1.11-1.58)	0.259/25.5	1.47(1.12-1.92)	0.397/0.0	1.29(1.13-1.47)	0.061/59.4

^a^ Number of studies;

^b^ P value of Q test for heterogeneity;

^c^ PB, population-based; HB, hospital-based;

^d^ HWE, Hardy-Weinberg equilibrium.

Of the eight studies included, four reported an association between the rs13181 polymorphism and risk of pancreatic cancer while the others did not. A significant association was observed between ERCC2 Lys751Gln polymorphism and susceptibility to pancreatic cancer in four genetic models [CC vs. AA: OR = 1.56, (95% CI: 1.28-1.90), P = 0.470, I2 = 0.0%; AC/CC vs. AA: OR = 1.20, (95% CI: 1.06-1.36), P = 0.396, I2 = 4.4%; CC vs. AC/CC: OR = 1.50; (95% CI: 1.24-1.81), P = 0.530, I2 = 0.0%; C vs. A: OR = 1.22, (95% CI:1.11-1.34), P = 0.159, I2 = 33.7%] (Figure 2A-2D, Table 2).

**Figure 3:**
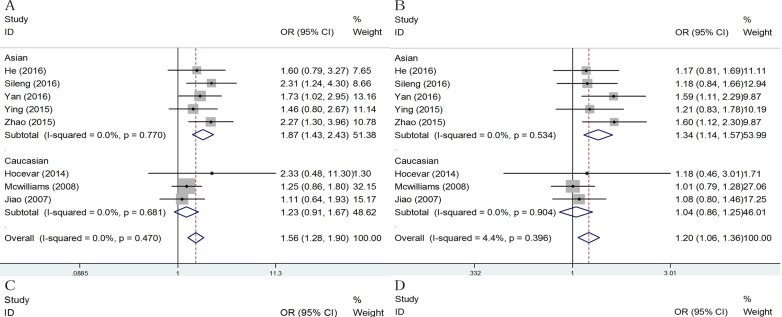
Subgroup analysis of ethnicity for ERCC2 rs13181 polymorphism and pancreatic cancer Statistical significance was observed in Asian population under four genetic models. A. Homozygote model: OR = 1.87, 95% CI: 1.43-2.43; B. Dominant model: OR = 1.34, 95% CI: 1.14-1.57;C. Recessive model: OR = 1.74; 95% CI: 1.35-2.25; D. Allele model: OR = 1.37, 95% CI:1.21-1.56.

Subgroup analysis by ethnicity showed that a significant association was identified in the Asian population [CC vs. AA: OR = 1.87, (95% CI: 1.43-2.43), P = 0.770, I2 = 0.0%; AC/CC vs. AA: OR = 1.34, (95%CI: 1.14-1.57), P = 0.534, I2 = 0.0%; CC vs. AC/CC: OR = 1.74; (95% CI: 1.35-2.25), P = 0.718, I2 = 0.0%; C vs. A: OR = 1.37, (95% CI:1.21-1.56), P = 0.507, I2 = 0.0%], but not in the Caucasian population (Figure 3A-3D, Table 2). In addition, stratified analysis by design of study showed a significant relationship in hospital-based studies (Supplementary Figure 1A-1D). Next, the Hardy-Weinberg equilibrium (HWE) of each study was taken into consideration. After eliminating studies whose distribution of genotype in controls deviated from HWE, the outcome remained statistically significant. (Supplementary Figure 2A-2D)

### Test of heterogeneity

The genetic heterogeneity between studies was evaluated based on all the five models and the data from the selected studies. By the Chi-squared-based Q-test, heterogeneity between studies was not identified in overall genetic models (P > 0.1). (Table 2)

### Sensitivity and publication bias analysis

**Figure 4:**
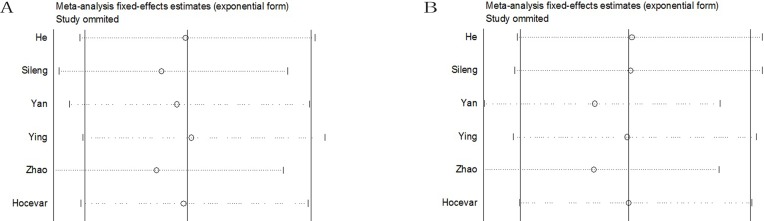
The sensitivity analysis of pancreatic cancer risk associated with ERCC2 rs13181 A > C polymorphism The pooled ORs were not influenced significantly by removal of each single study under four genetic models. A. Homozygote model; B. Dominant model; C. Recessive model; D. Allele model.

To further validate the robustness of the outcomes, we conducted sensitivity analyses by repeating the meta-analysis while sequentially omitting the studies included (one omitted each time) for every genotype model. The pooled ORs were not influenced significantly by removal of each single study under four genetic models (Figure 4A-4D), suggesting that the results of this meta-analysis were stable.

**Figure 5:**
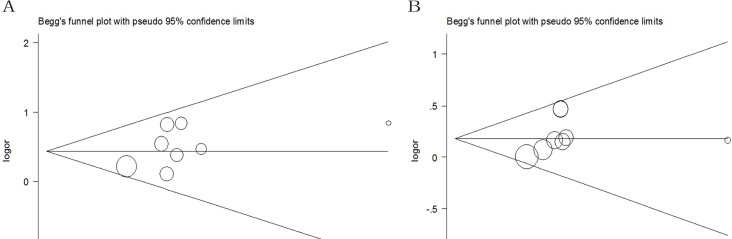
Begg's funnel plot of pancreatic cancer risk associated with ERCC2 rs13181 A > C polymorphism The funnel plots of A. homozygous, B. dominant, C. recessive and D. allele models are symmetrical inverted funnels, which suggests no significant publication bias.

To determine the possible publication bias of the literature, Begg's test and Egger's test were performed. The results of both Begg's and Egger's test showed no evidence of publication bias for ERCC2 Lys751Gln polymorphisms (Table 3). In addition, the funnel plots of the homozygous, dominant, recessive and allele models were symmetrical inverted funnels (Figure 5A-5D), which suggested no significant publication bias. The outcomes above indicated that the conclusions of our meta-analysis were stable and credible.

**Table 3:** The result of Begg and Egger's tests

Risk model	Egger' s test		Begg' s test	
	T statistic	*P* value	Z statistic	*P* value
Homozygous (CC vs. AA)	1.32	0.235	1.11	0.266
Dominant (AC/CC vs. AA)	1.00	0.355	1.11	0.266
Recessive (CC vs.AC/AA)	1.35	0.226	1.36	0.174
Allele (C vs. A)	1.50	0.185	1.11	0.266

## DISCUSSION

Pancreatic cancer is a highly lethal disease, for which mortality closely parallels incidence [2]. As a multi-factor disease, genetic mutation has been found to play a key role in its development and progression. Understanding the etiology and genetic background of pancreatic cancer is important for screening high-risk populations and promoting the development of molecular-targeted therapy.

Many previous genetic association studies on pancreatic cancer risk have focused on the effects of single nucleotide polymorphisms in ERCC2 gene. ERCC2, also known as Xeroderma pigmentosum D (XPD), is a key DNA repair gene in nucleotide excision repair (NER) pathway which could repair a wide variety of structurally DNA lesions, including cross-links, bulky adducts [19], thymidine dimers, oxidative DNA damage [20] and alkylating damage [21]. SNPs in exons of DNA repair genes can affect their protein activity, leading to differences of individual NER and DNA repair capacity (DRC) that may influence the susceptibility to pancreatic cancer. As the most frequently assessed variant, the ERCC2 Lys751Gln polymorphism is thought to be associated with many cancers [22–27]. In 2012, Sobti et al. [22] showed that ERCC2 mutations are associated with an increased risk of urinary bladder cancer in North Indian population. Meanwhile, Samson et al. [23] identified that polymorphisms of the ERCC2 gene might contribute to tumorigenesis in breast cancer among south Indian population. In addition, the ERCC2 gene could also increase the risk of hepatocellular carcinoma [24], acute lymphoblastic leukemia [25], lung cancer [26] and melanoma [27]. Recently, more studies have shown that the ERCC2 gene polymorphisms plays a key role in the tumorigenesis of pancreatic cancer [11–18]. However, the results remain inconsistent. In addition, there were no publically available GWAS databases and GWAS analysis which had evaluated this SNP and pancreatic cancer before. In order to elucidate the real association, this analysis was performed.

The current study is the first meta-analysis of the association between ERCC2 rs13181 and the risk of pancreatic cancer. Analysis among all subjects suggested a significant increase in the risk of pancreatic cancer associated with Gln/Gln or Lys/Gln genotype. All studies included were found homogeneous without any study disproportionately driving the combined estimates. In our meta-analysis, the genetic heterogeneity between the selected studies was evaluated, and no significant heterogeneity was observed in the homozygous, dominant, recessive and allele models.

Interestingly, the association remained statistically significant in subgroups (Asians, HB and the studies consistent with HWE) analyses. In subgroup analysis of ethnicity, our results showed that the C allele of rs13181 had a 1.22-fold risk of pancreatic cancer in overall populations, a 1.37-fold risk (95% CI 1.21-1.56) in Asian populations. Compared to the dominant and recessive models, the homozygous model showed the highest odds ratio in all populations (OR = 1.56, 95% CI 1.28-1.90) and in the Asian population (OR = 1.87, 95% CI 1.43-2.43). The differences between Asians and other races may be partly due to the different genetic backgrounds and environments or lifestyles.

Meta-analysis is a very powerful tool for analyzing cumulative data of studies where the individual sample sizes are small and the statistical power is low. However, there are still some limitations in the current meta-analysis. To begin with, our analysis was based on ORs estimated without adjustment for several potential confounding variables, because of a lack of information about cigarette smoking [28], chronic pancreatitis [29], diabetes [30] and a family history of pancreatic cancer [31], which are known to have significant effects on the development of pancreatic cancer. Secondly, more studies from all over the world should be performed to make our conclusions more persuasive, because our meta-analysis lacks studies in African populations. Finally, as a multi-factorial disease, pancreatic cancer results from complex interactions including a variety of genetic and environmental factors, suggesting pancreatic cancer susceptibility could not be influenced by any single gene. More researches exploring the influencing factors are required in the future.

In conclusion, our meta-analysis suggests that ERCC2 Lys751Gln polymorphism is a risk factor of pancreatic cancer for all of the ethnicities, and presence of this polymorphism in Asian population will increase their susceptibility to pancreatic cancer. However, additional larger and ethnically diverse studies are needed to further clarify the role of this polymorphism in the development of pancreatic cancer.

## MATERIALS AND METHODS

### Primary search strategy

We searched for relevant studies up to July 18, 2016 in both English and Chinese through PubMed, Web of Science, EMBSE and the China National Knowledge Infrastructure (CNKI) platforms database with the following terms and their combinations: “ERCC2 or XPD”, “polymorphism or variant”, “Lys751Gln”, “rs13181”. “K751Q” and “pancreatic cancer”, “pancreatic ductal adenocarcinoma”. To prevent the loss of any important data, we also identified additional investigations by screening the reference lists of key studies and reviews.

### Inclusion criteria and exclusion criteria

Studies involved had to satisfy the inclusion criteria: (a) case-control design was utilized; (b) researches focused on the association of ERCC2 Lys751Gln (rs13181) polymorphisms with the risk of pancreatic cancer; (c) sufficient data for estimating an odds ratio (OR) with 95% CI were available. The major exclusion criteria were as follows: (a) no obtainable genotype frequency data; (b) unpublished papers, dissertations, conference articles, reviews and duplication of publications (select the study in the latest and largest sample size); (c) studies designed as a case-case or case-only study.

### Data extraction

All the following information was extracted separately by two investigators (Y Wu, Z Lu) and recorded in a standardized form, including: first author's name, year of publication, ethnicity of each study population, source of controls, sample size, genotyping method, number of pancreatic cancer cases and controls, allele frequencies and genotype distributions of ERCC2 rs13181 in pancreatic cancer cases and controls respectively, and results of the Hardy-Weinberg equilibrium (HWE) test, as shown in Table 1. Inconsistencies were resolved by a discussion involving a senior investigator (K Jiang).

### Genetic model

The rs13181 polymorphism includes the two alleles A and C, of which C is the minor allele. C is assumed to be the high-risk allele and A is the low-risk allele. We selected the homozygous model (C/C vs. A/A), heterozygous model (A/C vs. A/A), dominant model (C/C + A/C vs. A/A), recessive model (C/C vs. A/C + A/A) and allele model (C vs A) for further meta-analysis.

### Statistical analysis

Stata software (version 12.0; StataCorp LP, College Station, TX) was applied in the whole statistical analyses. P values were all two-sided and regarded as statistically significant if less than 0.05.

To evaluate the strength of association between rs13181 and the risk of pancreatic cancer, the pooled odds ratios (ORs) with 95% CIs were calculated in the five genetic models. Meanwhile, between-study heterogeneity was evaluated using two methods including Cochran's Q-statistic and I2 = (Q-(k-1))/Q*100% statistic. Cochran's Q statistic approximately follows a χ2 distribution with k-1 degrees of freedom (k stands for the number of studies for analysis). A significant Q-statistic (P < 0.1) indicates heterogeneity among selected studies. I2 is a measure of heterogeneity and a statistic that indicates the percentage of variance in a meta-analysis that is attributable to study heterogeneity. The intervals including 0-25%, 25-50%, 50-75% and 75-100%, represent the low, moderate, large and extreme heterogeneity. The interval I2 > 50% indicates statistically significant heterogeneity. If heterogeneity P value was lower than 0.10, we considered the heterogeneity to be significant and random-effects model (The DerSimonian-Laird method) was used [32]. Otherwise, the fixed effects model (the Mantel-Haenszel method) was used [33]. Then, we conducted the subgroup analyses by collecting similar characteristics from the eligible studies, such as ethnicity (Asian and Caucasian), source of controls (population-based and hospital-based), and HWE (yes and no).

Sensitivity analysis was performed to assess the influence of individual studies on the pooled ORs, with the method of calculating the outcomes again by omitting one single study each time. Publication bias was evaluated with Begg's funnel plots and Egger's linear regression method and a P < 0.05 was set as the significance threshold [34]. HWE was checked by the goodness-of-fit chi-square test and a P < 0.05 was considered as a significantly selective bias [35].

## SUPPLEMENTARY MATERIALS FIGURES


