# Supplementary material for: Association between ERCC2 Lys751Gln polymorphism and the risk of pancreatic cancer, especially among Asians: evidence from a meta-analysis
Source: Oncotarget. 2017 Feb 16;8(30):50124–32. doi: 10.18632/oncotarget.15394 (PMC5564835; doi:10.18632/oncotarget.15394)
Supplement: Supplementary file 1 [file oncotarget-08-50124-s001.pdf]

Association between ERCC2 Lys751Gln polymorphism and the risk of pancreatic cancer, especially among Asians: evidence from a meta-analysis

Supplementary Material

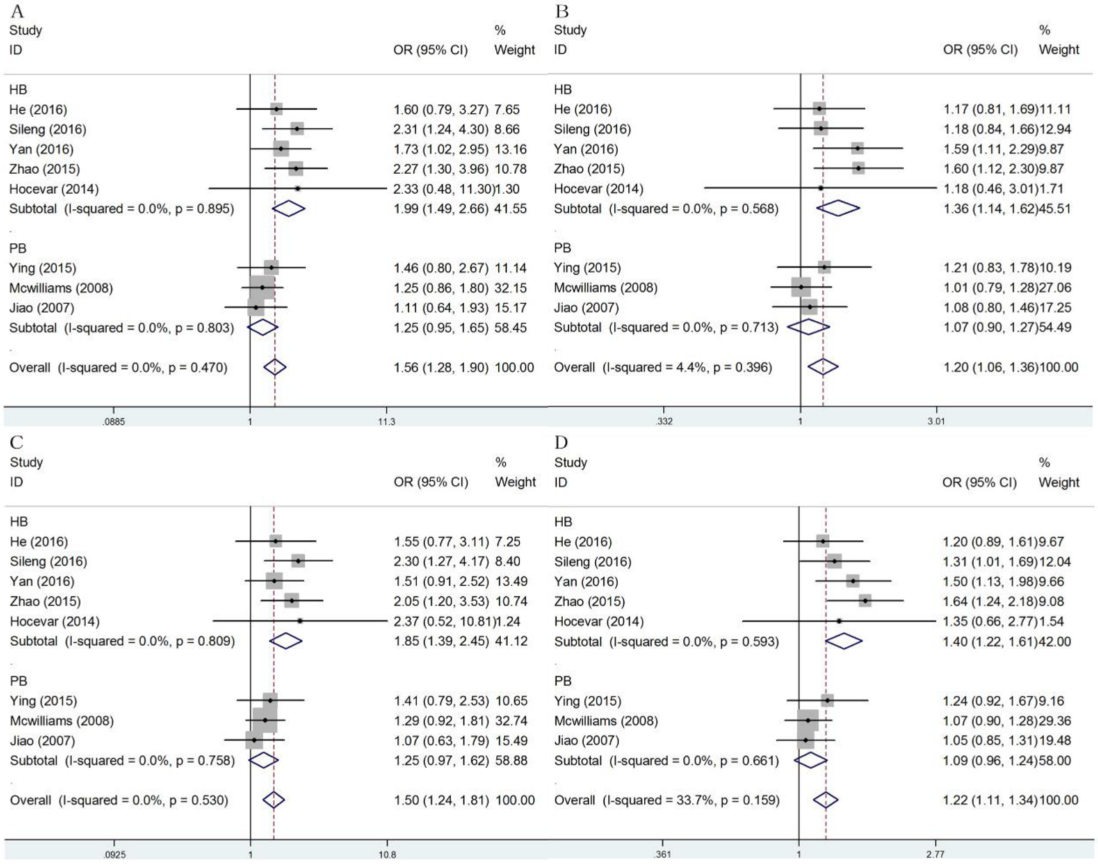

Supplementary Figure 1. Stratification analysis of source of controls for ERCC2 rs13181 polymorphism and pancreatic cancer. Statistical significance was observed in hospital-based studies under four genetic models. (A) Homozygote model; (B) Dominant model; (C) Recessive model; (D) Allele model.

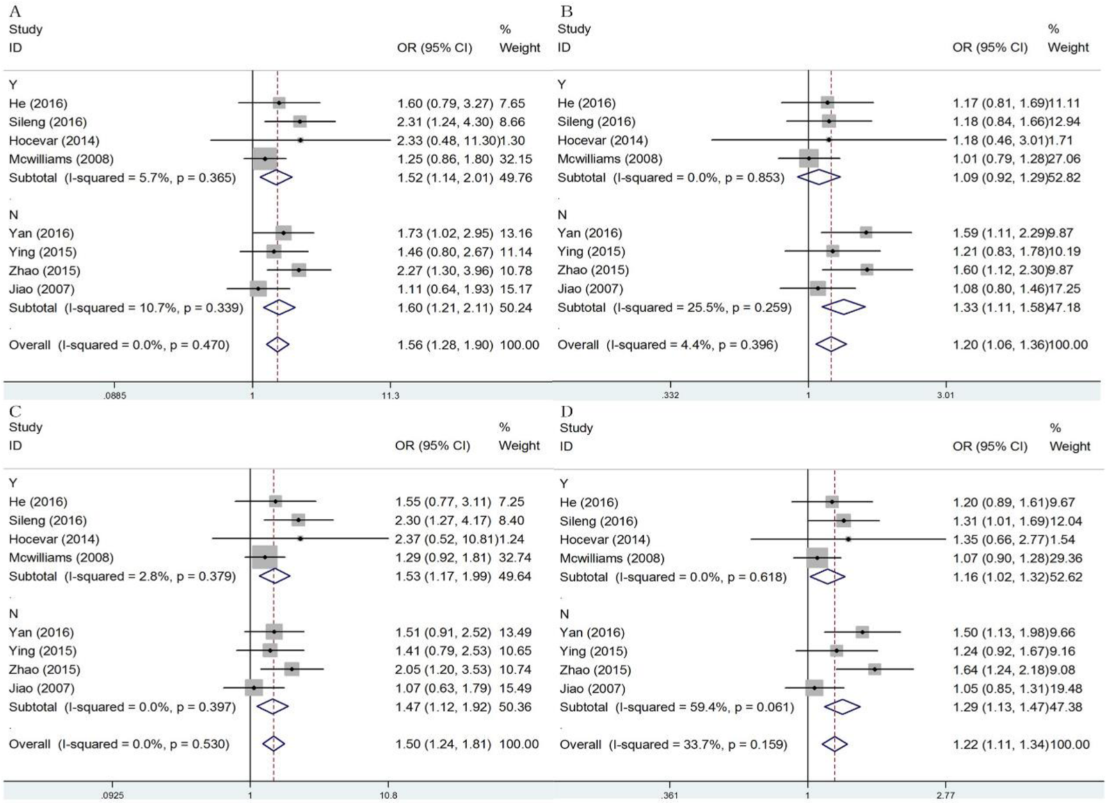

Supplementary Figure 2. Subgroup analysis of HWE for ERCC2 rs13181 polymorphism and pancreatic cancer. After excluding studies whose distribution of genotype in controls deviated from HWE, the outcome remained statistically significant. (A) Homozygote model; (B) Dominant model; (C) Recessive model; (D) Allele model.
